# Lung-tropic dual AAV-SP-C and microRNA gene therapy attenuates lung injury in mutant *Sftpc* mice

**DOI:** 10.1016/j.ymthe.2025.12.049

**Published:** 2025-12-24

**Authors:** Pauline Bardin, Liqun Xu, Yanlong Pei, Adithya Achuthan Vineeth, Kennedy Nangle, Shumei Zhong, Chanèle Cyr-Depauw, Arul Vadivel, Emily Martin, Timothy E. Weaver, Sneha Sitaraman, Sarah K. Wootton, Bernard Thébaud

**Affiliations:** 1 Sinclair Center for Regenerative Medicine, Ottawa Hospital Research Institute (OHRI) and Department of Cellular and Molecular Medicine, University of Ottawa, Ottawa, ON, Canada; 2 Department of Pathobiology, Ontario Veterinary College, University of Guelph, Guelph, ON, Canada; 3 Division of Pulmonary Biology, Cincinnati Children’s Research Foundation and Department of Pediatrics, University of Cincinnati College of Medicine, Cincinnati, OH, USA; 4 Division of Pulmonary & Sleep Medicine, Department of Pediatrics, Children’s Hospital of Philadelphia, Philadelphia, PA, USA; 5 Department of Pediatrics, Children’s Hospital of Eastern Ontario (CHEO) and CHEO Research Institute, Ottawa, ON, Canada

**Keywords:** adeno-associated virus, AAV, AAV6.2FF, surfactant protein C deficiency, I73T mutation, gene therapy, combinatorial therapy, lung-targeted gene therapy, inherited surfactant disorders

## Abstract

Mutations in the surfactant protein C (SP-C) gene (*SFTPC*) impair surfactant homeostasis, leading to respiratory distress in newborns or progressive interstitial lung disease. The most frequent mutation, I73T, results in the expression of a toxic dominant-negative form of SP-C, insinuating that for gene replacement therapy to be successful, suppression of the toxic form of the SP-C protein may also be necessary. Here, we capitalized on our rationally designed lung-tropic adeno-associated virus (AAV)6.2FF vector to develop a combinatorial gene therapy approach for treating SP-C disorders. In I73T-knockin mice exhibiting decreased *Sftpc* expression and toxic prosurfactant protein C (proSP-C) accumulation resulting in focal airspace enlargement, gene replacement therapy via airway delivery of AAV6.2FF expressing SP-C restored wild-type (WT) *Sftpc* and mature SP-C protein expression while significantly improving lung function and focal airspace enlargement. Next, we developed a dual-function AAV6.2FF to express functional SP-C and suppress the toxic 173T *SFTPC* gene (AAV-SPC-miR). The dual-function AAV6.2FF-SP-C-miR decreased endogenous *Sftpc* expression, restored WT *Sftpc*, re-expressed the mature SP-C protein without significant proSP-C protein accumulation, and attenuated airspace enlargement. These findings suggest that combination gene therapy is feasible and represents a promising tool for treating SP-C deficiencies and *SFTPC* mutation-linked lung diseases in humans.

## Graphical abstract

**Figure:**
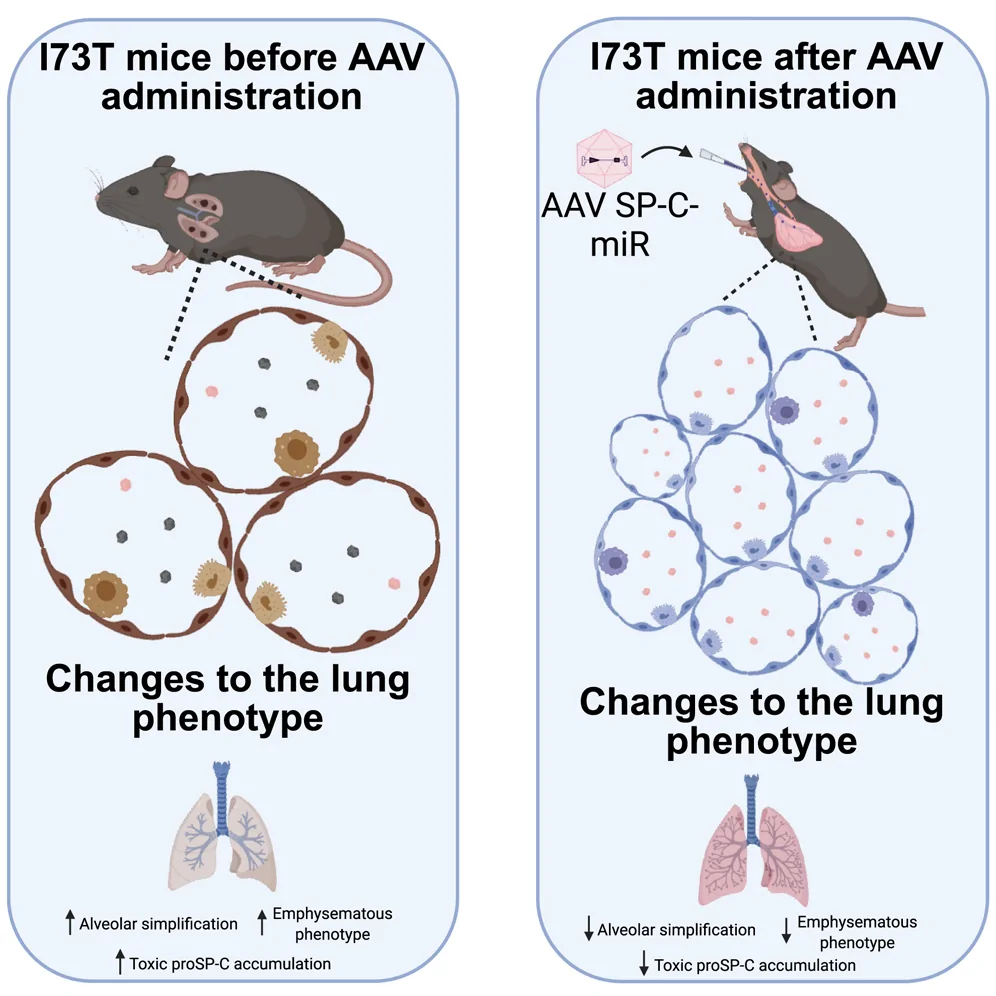

## Teaser

Thébaud and colleagues demonstrate that a combinatorial gene therapy using a lung-tropic AAV6.2FF vector can both restore functional surfactant protein C expression and suppress the toxic I73T mutant form. This dual-function approach offers a promising strategy for treating SFTPC mutation-linked neonatal and progressive interstitial lung diseases.

## Introduction

Pulmonary surfactant, a protein and lipid mix produced by type II alveolar epithelial cells (AECIIs), reduces alveolar surface tension and prevents atelectasis. Family-based studies have identified causal genetic variants linked to the impaired production and function of pulmonary surfactant. Many of these genetic variants are described in the pediatric population as part of the broader spectrum of childhood interstitial lung diseases (chILDs). chILDs can present with respiratory distress due to surfactant protein (SP) deficiency caused by mutations in *SFTPB*, *SFTPC* (encoding for SPs), *ABCA3* (packaging and secretion of SPs), and *NKX2.1* (transcription factor regulating SP expression in lung tissue).^1^ Mutations in the *SFTPC* gene leading to SP-C deficiency can result in a wide spectrum of clinical manifestations, from acute respiratory distress in newborns to progressive ILD in adulthood.^2^

We have previously demonstrated the potential of gene replacement using a rationally engineered adeno-associated virus (AAV) vector to treat SP-B deficiency.^3^ This AAV6.2FF displays high lung tropism and preferentially targets AECIIs (78.8%), leading to rapid expression of the wild-type (WT) SP-B transgene. A single AAV6.2FF-SP-B intratracheal administration significantly improved lung function and median survival in SP-B-deficient mice. Our compelling findings strongly suggest this AAV vector as a promising treatment for chILDs associated with surfactant deficiencies.

Mutations in *SFTPC* are present in an autosomal-dominant manner with variable penetrance and severity. The most frequent mutation, I73T (g.1286T>C), is a toxic gain-of-function mutation that leads to abnormal processing of prosurfactant protein C (proSP-C) to mature SP-C, resulting in impaired surfactant production and symptoms of ILD. In this study, we aimed to use a recently described I73T SP-C knock-in (KI) mouse model^4^ to test the feasibility and efficacy of an AAV gene therapy to deliver a combination of a targeted knockdown of the endogenous toxic protein product using microRNAs and simultaneously provide a gene replacement with a codon-optimized, miRNA-resistant copy of SP-C cDNA for gene replacement to reduce/reverse lung injury in I73T KI mice. The I73T SPC-KI mouse model demonstrates abnormal trafficking and processing of SP-C(I73T) proprotein, characterized by accumulation near cell surfaces, impaired processing into mature SP-C peptide, and altered expression of proteins involved in vesicular trafficking and mitochondrial function.^5^ It was shown that the toxic proproteins were not identified and were eliminated through the ERAD pathway. These defects lead to alveolar simplification and mitochondrial dysfunction in AECIIs that successfully recapitulate key aspects of the human *SFTPC* I73T-related disease, particularly the mis-trafficking and misprocessing of the pathogenic variant of the SP-C protein.^4^

## Results

### I73T mice showed focal airspace enlargement associated with the accumulation of proSP-C

**Figure 1:**
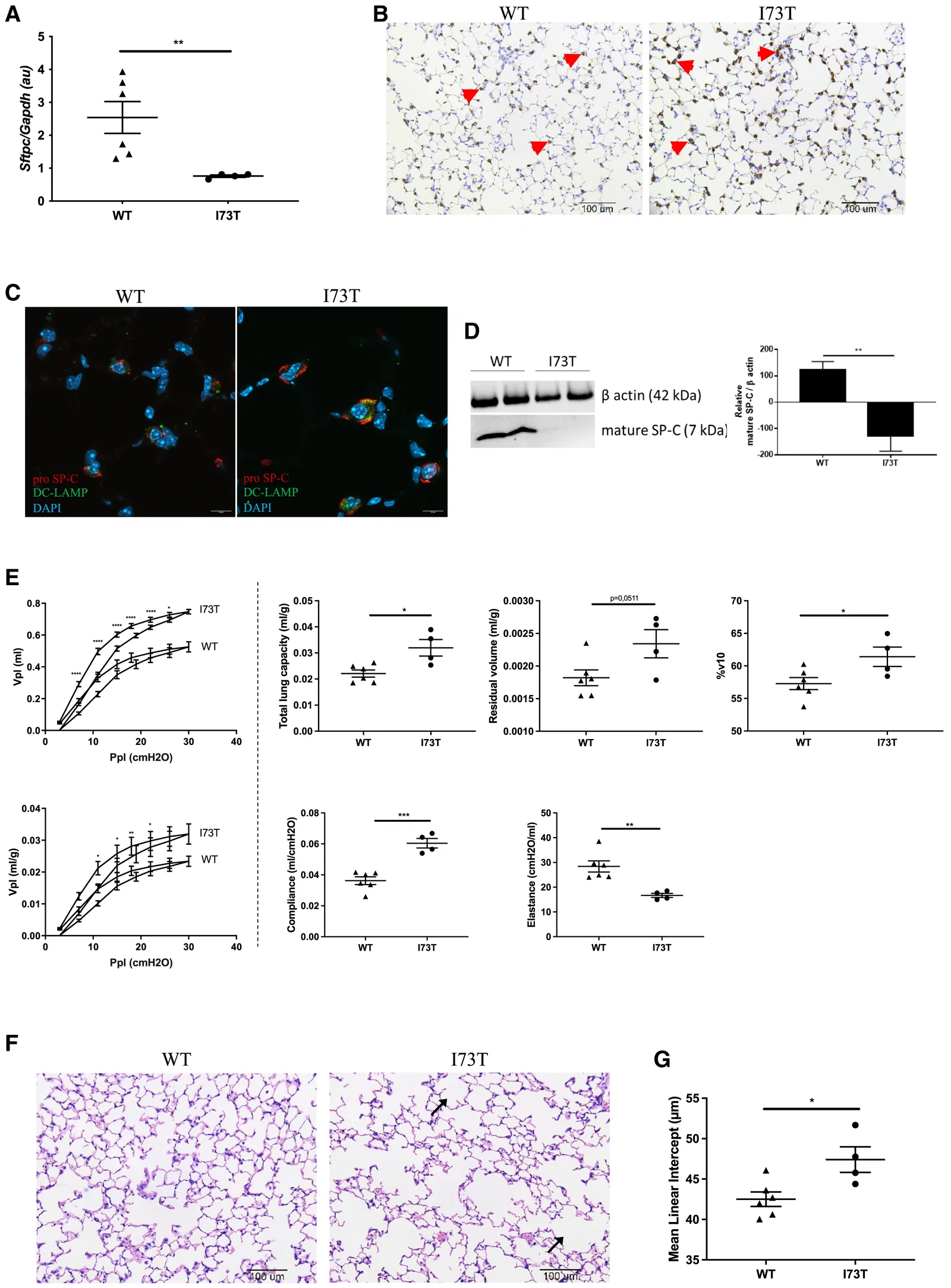
I73T-knockin mouse model characterization (A) Quantification of *Sftpc* endogenous expression in lung tissue homogenate from WT (*n* = 6) and I73T (*n* = 4) mice, relative to *Gapdh* (∗∗*p* = 0.0026). (B) Immunohistochemistry of proSP-C (red arrows) on lung sections of WT and I73T. (C) Representative fluorescent immunohistochemistry images showing coexpression of proSP-C (red), DC-LAMP (dendritic cell - lysosomal associated membrane protein) (green), and DAPI (blue) in the airway epithelium of WT (left) and I73T (right) mouse lungs. Scale bars: 10 μm. (D) Representative western blot and quantification of mature SP-C (∗∗*p* = 0.0015) protein expression levels relative to β-actin in lung tissue homogenate from WT (*n* = 6) and I73T (*n* = 4) mice. (E) Pressure-volume (PV) curve and body-weight-adjusted PV loops. PV loops are shown as the mean volume of all animals within one group at each pressure point (WT, *n* = 6; I73T, *n* = 4). Total lung capacity (TLC; ∗*p* = 0.0114), %v10 (∗*p* = 0.0378), and residual volume (*p* = 0.0511) are corrected for body weight. Compliance (∗∗*p* = 0.0003) and elastance (∗∗*p* = 0.0037) are shown. (F) Representative images of lung histology stained with H&E from WT and I73T mice. Focal airspace enlargement (arrows) is observed in injured lungs. (G) Graph depicting the mean linear intercept (MLI) based on H&E-stained slides from WT (*n* = 6) and I73T (*n* = 4, ∗*p* = 0.0194). The sex of the animals in WT (*n* = 6, 3 males and 3 females) and I73T (*n* = 4, 2 males and 2 females) mice is shown. An unpaired *t* test was used to determine the significance between the two conditions. Data are presented as mean ± SEM.

**Figure 2:**
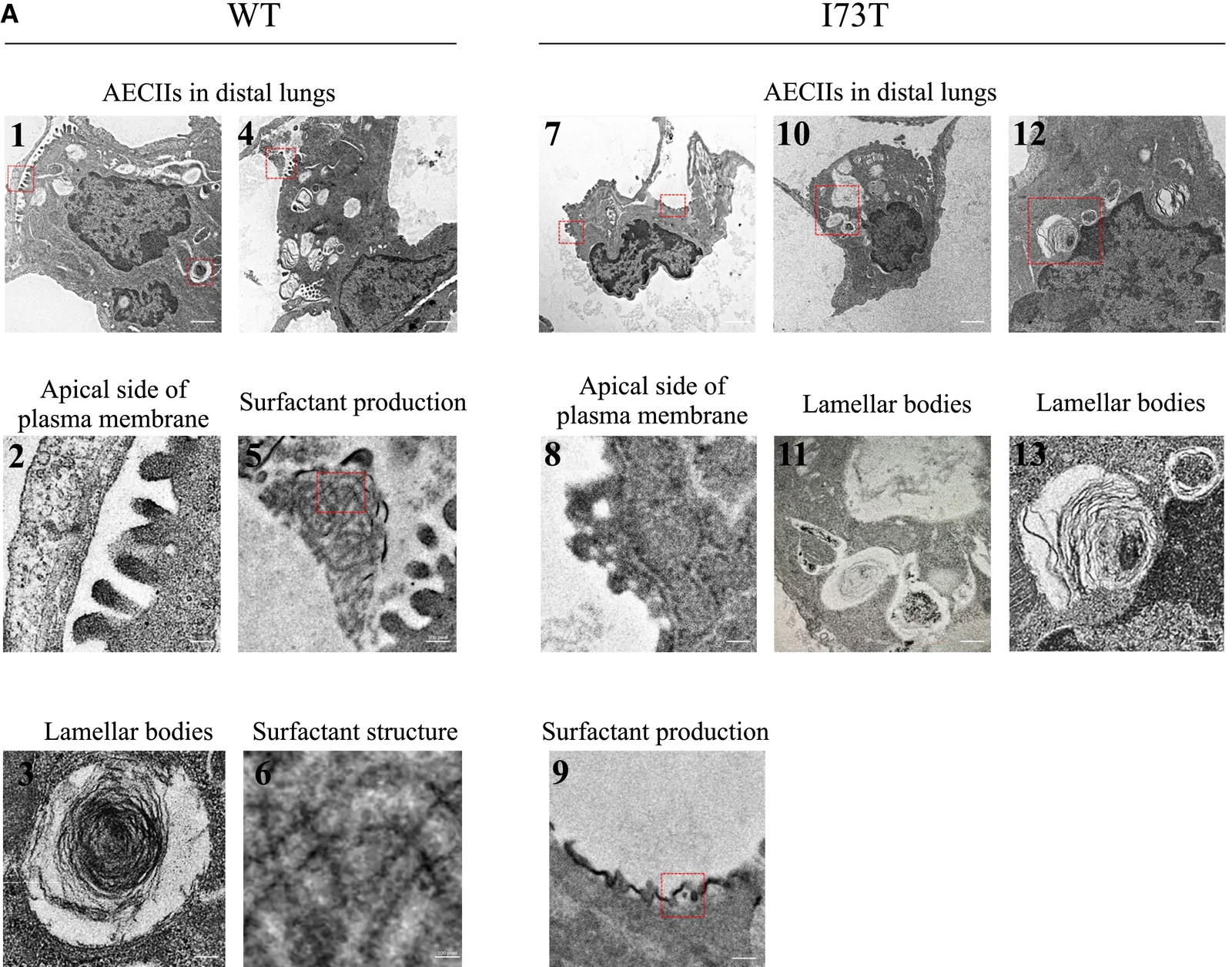
Ultrastructural characterization of AECII in the I73T-knockin mouse model Transmission electron micrographs showing type II alveolar epithelial cells (AECIIs) in the distal lung from WT (1 and 4) and I73T (7, 10, and 12) mice. Scale bars: 2 μm; magnification: ×5,000. Ultrastructure of the apical side of the plasma membrane (2 and 8), lamellar bodies (3, 11, and 12), and surfactant production (5 and 9) with the meshwork of tubular myelin produced in the alveolar airspace. Scale bars: 500 nm. The ultrastructure of the surfactant is shown in more detail (6). Scale bars: 100 nm. The region enclosed by the red box in the top image is shown at a higher magnification in the corresponding bottom image. In box 9, the red box highlights the area of interest.

To assess the natural history of *SFTPC*-related disease and evaluate candidate gene therapies, an I73T mutation was inserted into the mouse *Sftpc* locus as recently described.^4^ Here, these mice were used to assess the impact of a novel AAV gene therapy on various lung parameters. I73T mice with the KI mutation showed a significant decrease in lung *Sftpc* gene expression (Figure 1A) and proSP-C accumulation (Figures 1B and 1C), suggestive of a processing defect leading to an accumulation of pro-SPC, as observed in human patients with this pathogenic variant, and a significant decrease in mature SP-C protein expression (Figures 1D and S3A) compared to the lungs of WT mice at 8- to 10 weeks of age. Although mutant allele expression was reduced, proSP-C protein levels exceeded those of the WT (Figures 1B and 1C). In addition, lung sections from I73T mice showed marked AECII hyperplasia, with a notably higher abundance of proSP-C and ABCA3-positive cells compared to WT lungs (Figures 1B and S1A). In contrast to the intracellular punctate localization of the WT SP-C proprotein in AT2 cells, the SP-C(I73T) proprotein exhibited intense accumulation near the cell surface (Figure 1D). I73T mice did not show a difference in body weight compared to the WT (Figure S1B). I73T mice displayed impaired lung function characterized by an increase in total lung capacity, %v10, and compliance and a decrease in elastance compared to the WT, consistent with an emphysematous phenotype^6^ (Figure 1E). Accordingly, I73T mice developed focal airspace enlargement (Figure 1F), as quantified by structural lung morphometry using the mean linear intercept (MLI) method, which is a quantitative metric that gives a simple and direct notion of the size of structural lung units and changes to it.^7^ MLI was significantly increased compared to the WT (Figure 1G). I73T mice did not develop spontaneous fibrosis, as demonstrated by the lack of difference in the Ashcroft-Hübner (AH) modified score (Figure S1C), right lung weight (Figure S1D), Sirius red staining (Figure S1E), and expression of extracellular matrix genes such as *Col1a1* and *Col1a2* (Figure S1F) compared to the WT mice. Similarly, no difference was observed in *Tgfb1* (marker of fibrosis) and *Atf6* (marker of endoplasmic reticulum stress) expression (Figure S1F). Similar to human findings, the ultrastructure of the apical side of the plasma membrane (Figure 2, box 8) and lamellar bodies (Figure 2, boxes 11 and 13) was altered, and surfactant production with the meshwork of tubular myelin (Figure 2, box 9) produced in the alveolar airspace was decreased or missing in I73T compared to WT mice (Figure 2, box 5).

### *In vitro* validation of AAV vector constructs

**Figure 3:**
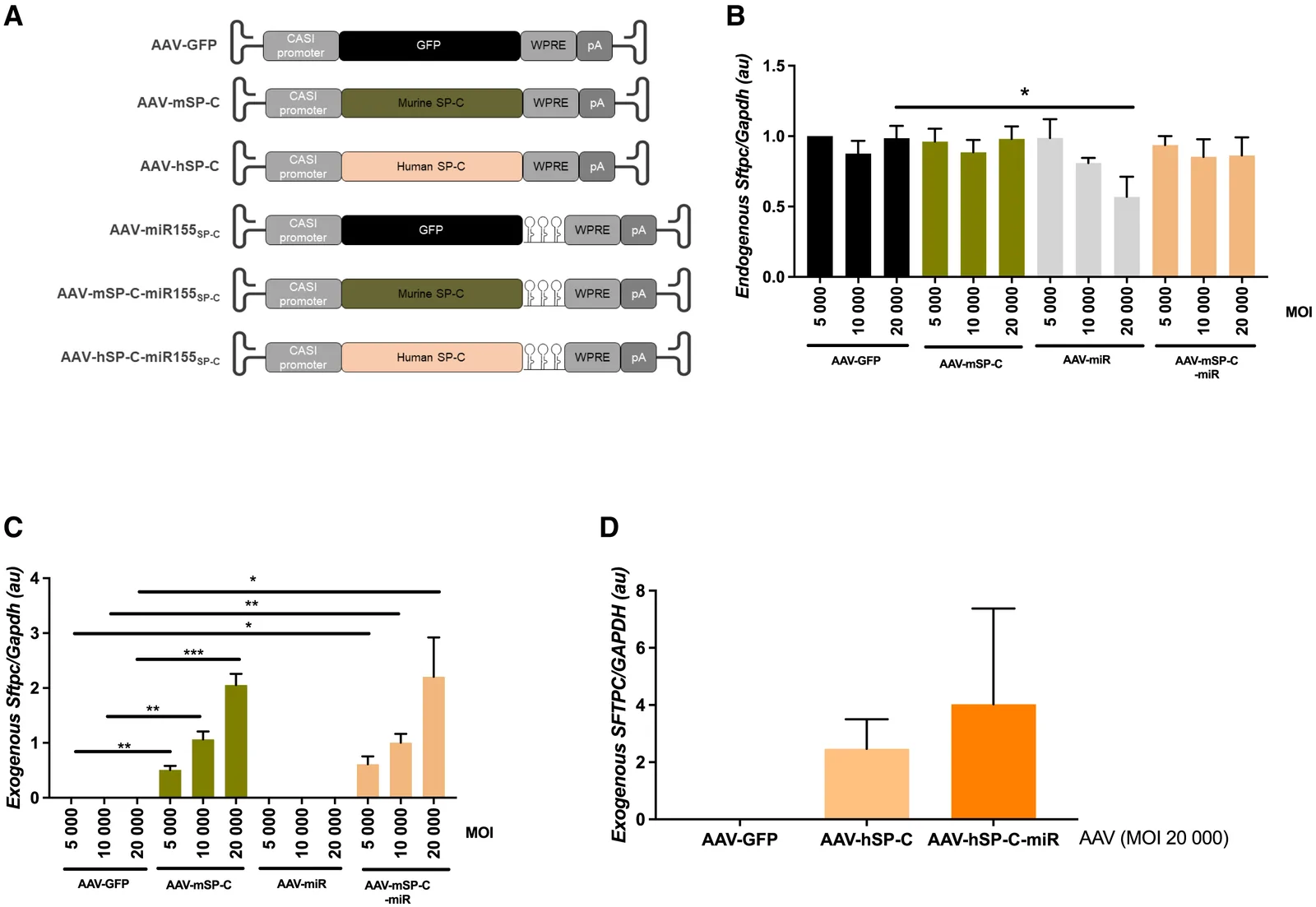
Schematic representation of AAV vector constructs and *in vitro* tests (A) All adeno-associated virus (AAV) genomes are flanked by AAV2 inverted terminal repeats (ITRs) and contain the ubiquitous CASI promoter, a woodchuck hepatitis virus post-transcriptional regulatory element (WPRE), and an SV40 poly(A) (p(A)) signal. AAV-GFP encodes the EGFP and is used as a control for reference during the experiments. AAV-mSPC and AAV-hSPC vectors encode the murine and human surfactant protein C genes, respectively. AAV-miR-155_SPC_ contains the *EGFP* gene followed by a miR-155 cassette encoding three different miRNAs against endogenous murine *Sftpc* (miR-155_SP-C_). AAV-mSPC-miR-155_SPC_ and AAV-hSPC-miR-155_SP-C_ encode miR-155_SP-C_-resistant murine and human *Sftpc* and *SFTPC* genes, respectively, followed by the miR-155 cassette against the endogenous *Sftpc* gene. (B) Quantification of the *Sftpc* endogenous expression in MLE12 cells transduced for 96 h with AAV-GFP (*n* = 7), AAV-mSP-C (*n* = 5), AAV-miR (*n* = 3, ∗*p* = 0,0330), and AAV-mSP-C-miR (*n* = 3) at different MOIs (5,000–20,000), relative to *Gapdh* (not significant). (C) Quantification of the *Sftpc* exogenous expression in MLE12 cells transduced for 96 h with AAV-GFP (*n* = 7), AAV-mSP-C (*n* = 5, ∗∗*p* = 0.0022 and 0.0017, respectively, and ∗∗∗*p* = 0.0005), AAV-miR (*n* = 3), and AAV-mSP-C-miR (*n* = 3, ∗*p* = 0.0125, ∗∗*p* = 0.0032, and ∗∗∗*p* = 0.0370) at different MOIs (5,000–20,000) relative to *Gapdh*. (D) Quantification of the *SFTPC* exogenous expression in HEK293 cells transduced for 96 h with AAV-GFP (*n* = 8), AAV-hSP-C (*n* = 8), and AAV-hSP-C-miR (*n* = 7) at different MOIs (5,000–20,000) relative to *GAPDH* (not significant). One-way and two-way ANOVA tests were used to determine significance. Data are presented as mean ± SEM.

To rescue cytotoxic proSP-C proprotein accumulation in I73T mice, we developed several AAV constructs that were packaged into the lung-tropic AAV6.2FF capsid (Figure 3A). These include vectors designed to express functional murine or human SP-C as well as vectors that expressed functional SP-C (codon optimized to be miRNA resistant) in combination with miRNA cassettes designed to suppress endogenous I73T mutant SP-C (Figure 3A). *In vitro* RT-qPCR analysis in MLE-12 cells showed that AAV-mSP-C did not alter the expression of the endogenous *Sftpc* (Figure 3B). In parallel, it led to the re-expression of a healthy codon-optimized exogenous *Sftpc* transcript (Figure 3C). AAV-miR significantly decreased endogenous *Sftpc* expression (Figure 3B) at a multiplicity of infection (MOI) of 20,000 but not at lower MOIs (5,000 and 10,000). It is important to note that high MOIs of AAV (e.g., 100,000) are often required for efficient transduction of cells *in vitro*.^8^ The combinatorial construct AAV-mSP-C-miR showed no modulation of endogenous *Sftpc* expression (Figure 3B) and showed a re-expression of a healthy codon-optimized exogenous version compared to AAV-GFP (Figure 3C). Similar results were observed with AAV-hSP-C and AAV-hSP-C-miR (Figure 3D), where both constructs increased human codon-optimized exogenous *SFTPC* expression at an MOI of 20,000.

### AAV gene therapy restores expression of mature SP-C and attenuates airspace enlargement

**Figure 4:**
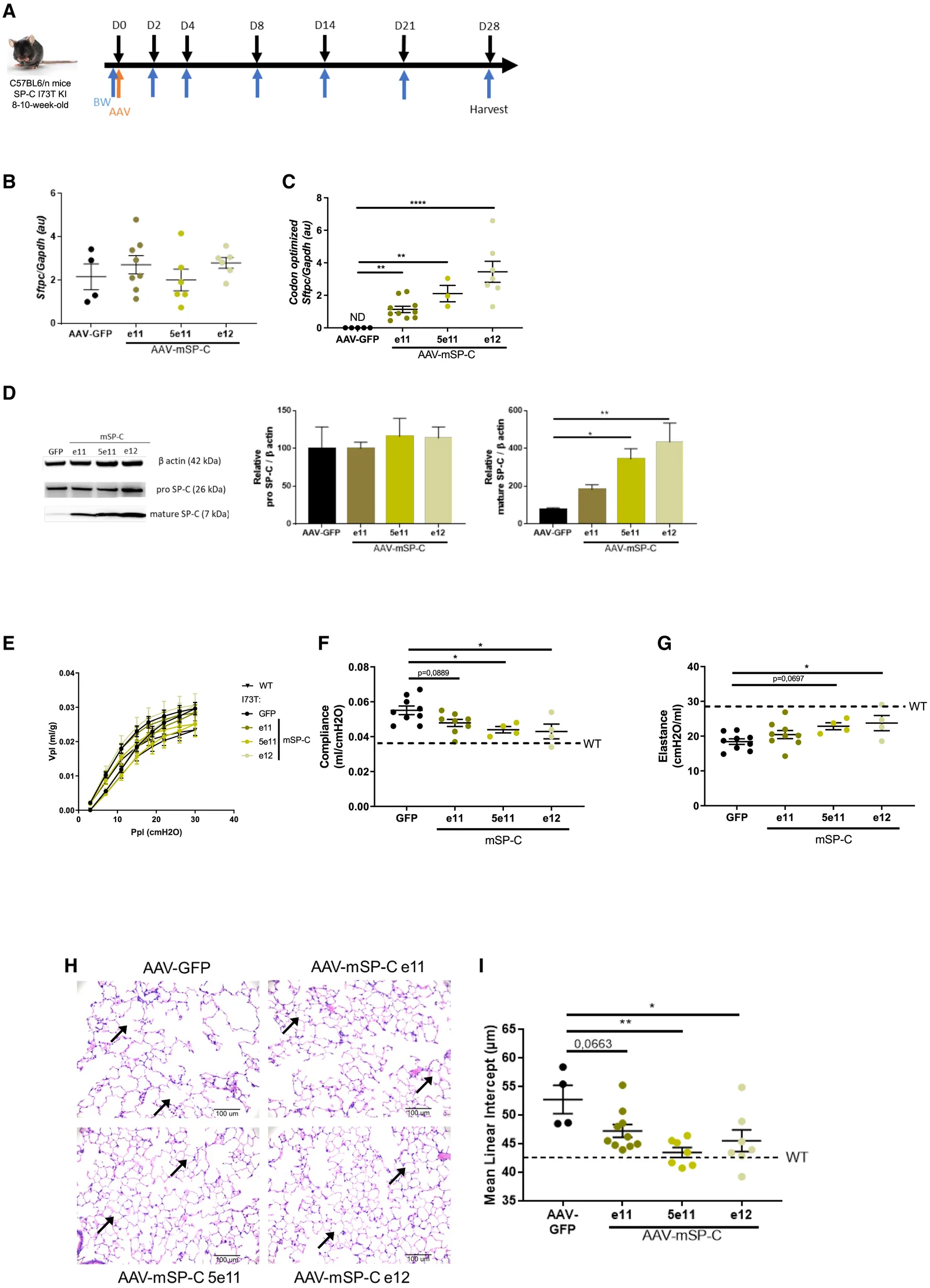
AAV-SP-C restores SP-C expression and attenuates focal airspace enlargement in I73T mice (A) Experimental design. (B) Quantification of *Sftpc* endogenous expression in lung tissue homogenate from I73T treated with AAV-GFP (*n* = 4) and AAV-mSP-C at different concentrations—e^11^ vg (*n* = 8, 4 males and 4 females), 5e^11^ vg (*n* = 6, 3 males and 3 females), and e^12^ vg (*n* = 6, 3 males and 3 females)—for 28 days, relative to *Gapdh*. (C) Quantification of *Sftpc* exogenous expression in lung tissue homogenate from I73T treated with AAV-GFP (*n* = 5) and AAV-mSP-C at different concentrations—e^11^ vg (*n* = 10, ∗∗*p* = 0.0014), 5e^11^ vg (*n* = 3, ∗∗*p* = 0.0012), and e^12^ vg (*n* = 7, ∗∗∗∗*p* = 0.0001)—for 28 days, relative to *Gapdh*. (D) Representative western blot and quantification of proSP-C (not significant) and mature SP-C protein expression levels in lung tissue homogenate from I73T treated with AAV-GFP (*n* = 3) and AAV-mSP-C at different concentrations—e^11^ vg (*n* = 10, 5 males and 5 females), 5e^11^ vg (*n* = 7, 3 males and 4 females, ∗*p* = 0.0422), and e^12^ vg (*n* = 6, 3 males and 3 females, ∗∗*p* = 0.0072)—for 28 days, relative to β-actin. (E) Pressure-volume (PV) curve and body-weight-adjusted PV loop. PV loops are shown as the mean volume of all animals within one group at each pressure point. (F) Compliance (GFP, *n* = 9, 4 males and 5 females; mSP-C e^11^, *n* = 8, 4 males and 4 females, *p* = 0.0999; 5e^11^, *n* = 4, 2 males and 2 females, ∗*p* = 0.0290; and e^12^, *n* = 4, 2 males and 2 females, ∗*p* = 0.0167). (G) Elastance (GFP, *n* = 9, 4 males and 5 females; mSP-C e^11^, *n* = 8, 4 males and 4 females, *p* = 0.3841; 5e^11^, *n* = 4, 2 males and 2 females, *p* = 0.0697; and e^12^, *n* = 4, 2 males and 2 females, ∗*p* = 0.054). (H) Representative images of lung histology stained with H&E from I73T mice treated with (A) AAV-GFP and AAV-mSP-C at different concentrations—e^11^, 5e^11^, and e^12^ vg—for 28 days. Focal airspace enlargement (arrows) is diminished in treated lungs. (I) Graph depicting the MLI assessed on H&E-stained slides from I73T treated with (A) AAV-GFP (*n* = 4, 2 males and 2 females) and AAV-mSP-C at different concentrations—e^11^ vg (*n* = 10, 5 males and 5 females, *p* = 0.0663), 5e^11^ vg (*n* = 7, 4 males and 3 females, ∗∗*p* = 0.0048), and e^12^ vg (*n* = 7, 4 males and 3 females,∗*p* = 0.0337)—for 28 days. One-way and two-way ANOVA tests were used to determine significance. Data are presented as mean ± SEM.

*In vivo*, in WT mice, AAV-miR showed decreased proSP-C protein expression compared to AAV-luciferase, which was used as a control (Figures S2A and S2B). Different doses of AAV-mSP-C vector (e^11^, 5e^11^, and e^12^ vg/mouse) were delivered through a single oropharyngeal administration in I73T mice (Figure 4A). AAV-mSP-C did not affect body weight or right lung weight (Figures S4A and S4B). Hereafter, “exogenous expression” denotes the healthy codon-optimized *Sftpc*, and “endogenous expression” denotes the mutant *Sftpc*. As expected, AAV-mSP-C increased exogenous *Sftpc* expression (Figure 4C) without modulating endogenous *Sftpc* expression (Figure 4B), consistent with the data obtained *in vitro*. The highest doses of AAV-mSP-C (5e^11^ and e^12^ vg/mouse) significantly increased mature SP-C protein, without inducing increased proSP-C protein expression (Figures 4D and S3B). The exogenous SP-C expressed by the AAV was processed effectively into mature SP-C without any misprocessing, preventing further accumulation of proSP-C protein. At the functional level, AAV-mSP-C significantly improved lung compliance (5e^11^ and e^12^ vg/mouse) (Figure 4F) and elastance (e^12^ vg/mouse dose only) (Figure 4G). At the structural level, AAV-mSP-C (5e^11^ and e^12^ vg/mouse) reduced focal airspace enlargement (Figure 4H) and significantly decreased the MLI to WT levels (Figure 4I).

**Figure 5:**
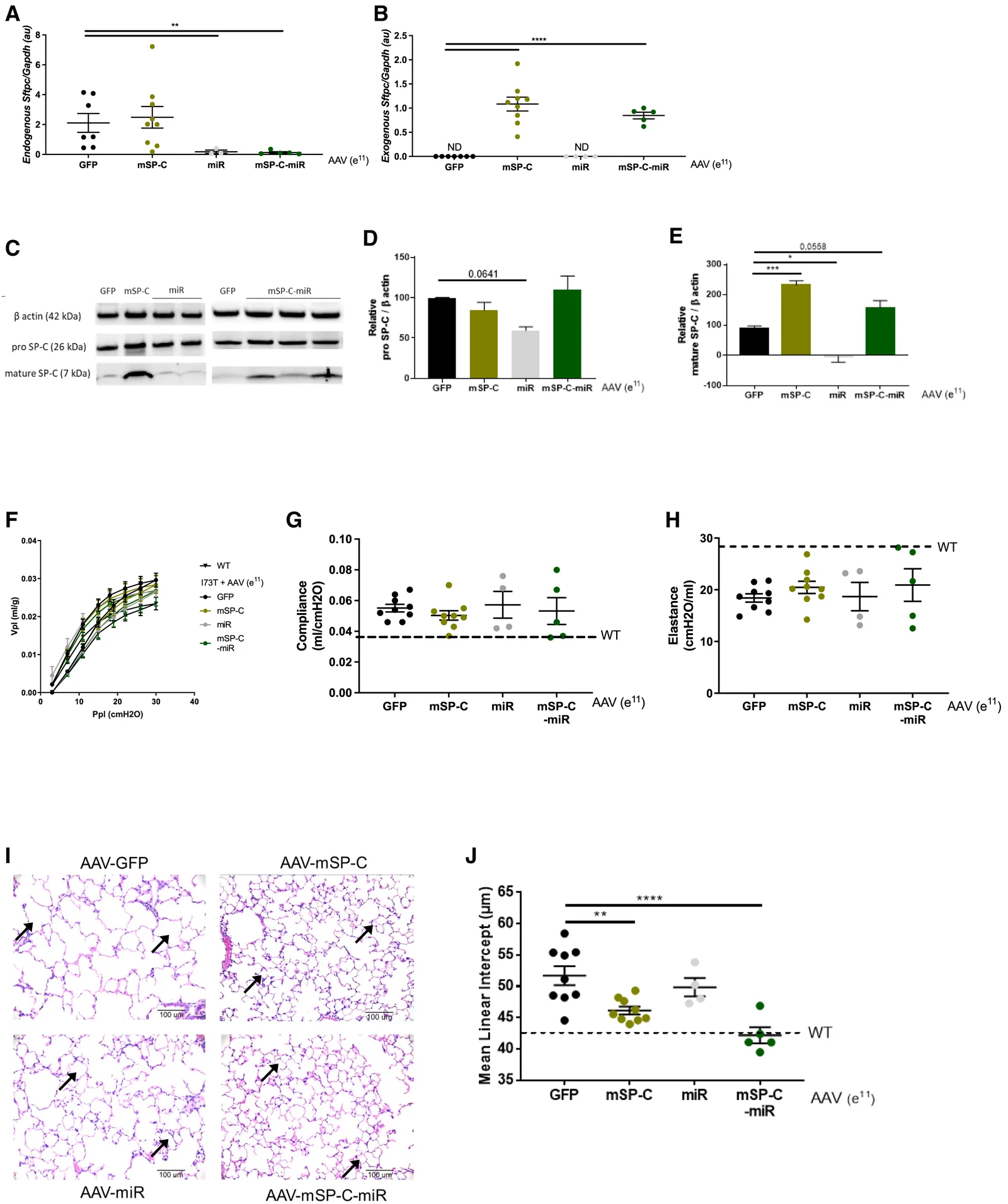
AAV-SP-C-miR restores SP-C expression and attenuates focal airspace enlargement in I73T mice (A) Quantification of endogenous *Sftpc* expression in lung tissue homogenate from I73T treated with AAV-GFP (*n* = 7, 3 males and 4 females), AAV-mSP-C (*n* = 9, 5 males and 4 females), AAV-miR (*n* = 4, 2 males and 2 females), and AAV-mSP-C-miR (*n* = 5, 2 males and 3 females) (∗∗*p* = 0.0051 and 0.0025, respectively) at e^11^ vg for 28 days, relative to *Gapdh*. (B) Quantification of exogenous *Sftpc* in lung tissue homogenate from I73T treated with AAV-GFP (*n* = 7, 4 males and 3 females), AAV-mSP-C (*n* = 9, 5 males and 4 females, ∗∗∗∗*p* = 0.0001), AAV-miR (*n* = 4, 2 males and 2 females, *p* = 0.9999), and AAV-mSP-C-miR (*n* = 5, 2 males and 3 females, ∗∗∗∗*p* = 0.0001) at e^11^ vg for 28 days, relative to *Gapdh*. (C) Representative western blot images and quantification of proSP-C (not significant) (D) and mature SP-C (E) protein expression levels in lungs from I73T treated with GFP (*n* = 4, 2 males and 2 females), mSP-C (*n* = 4, 2 males and 2 females, ∗∗∗*p* = 0.0006), miR (*n* = 4, 2 males and 2 females, ∗*p* = 0.0273), and mSP-C-miR (*n* = 5, 2 males and 3 females, *p* = 0.0558) at e^11^ vg for 28 days, relative to β actin. (F) Pressure-volume (PV) curve and body-weight-adjusted PV loop. PV loops are shown as the mean volume of all animals within one group at each pressure point (GFP, *n* = 9, 4 males and 5 females; mSP-C, *n* = 9, 4 males and 5 females; miR, *n* = 4, 2 males and 2 females; mSP-C-miR, *n* = 5, 3 males and 2 females; and e^11^, not significant). (G) Compliance (GFP, *n* = 9; mSP-C, *n* = 9; miR, *n* = 4; mSP-C-miR, *n* = 5; and e^11^, not significant; the sex of the animals in each group is the same as from the PV loops). (H) Elastance (GFP, *n* = 9; mSP-C, *n* = 9; miR, *n* = 4; mSP-C-miR, *n* = 5; and e^11^, not significant; the sex of the animals in each group is the same as from the PV loops). (I) Representative images of lung histology stained with H&E from I73T mice. Focal airspace enlargement (arrows) is diminished in treated lungs. (J) Graph depicting the MLI assessed on H&E-stained slides from I73T treated with GFP (*n* = 9), mSP-C (*n* = 9, ∗∗*p* = 0.0045), miR (*n* = 4, *p* = 0.6807), and mSP-C-miR (*n* = 5, ∗∗∗∗*p* = 0.0001) at e^11^ vg for 28 days (the sex of the animals in each group is the same as from the PV loops). One-way and two-way ANOVA tests were used to determine significance. Data are presented as mean ± SEM.

Next, to determine if knockdown of endogenous *Sftpc* I73T (using AAV-miR) would be sufficient to prevent the toxicity caused by the mutant protein or if a combinatorial approach of gene replacement with knockdown of the mutant protein (AAV-mSP-C-miR) would have an added therapeutic benefit, AAV-mSP-C, AAV-miR, and AAV-mSP-C-miR were delivered *in vivo* through a single oropharyngeal administration (e^11^ vg/mouse) (Figure 4A). The different AAV constructs did not affect body weight or right lung weight (Figures S4C and S4D). AAV-miR alone significantly silenced endogenous *Sftpc* expression (Figure 5A). The combined AAV-mSP-C-miR decreased endogenous *Sftpc* expression and increased exogenous *Sftpc* expression (Figure 5B). AAV-miR reduced accumulated proSP-C protein excess in I73T mice, while AAV-mSP-C-miR did not significantly modulate proSP-C protein (Figures 5C, 5D, and S3C), but both re-expressed the mature SP-C protein (Figures 5C, 5E, and S3C). At the functional level, AAV-mSP-C-miR showed no improvement (Figures 5F–5H). At the dose of e^11^ vg/mouse, AAV-miR could not modulate the MLI, whereas the combined AAV-mSP-C-miR construct showed a reduction in focal airspace enlargement (Figure 5I) and significantly decreased the MLI to WT levels (Figure 5J), equivalent to the effect seen with the 5e^11^ vg/mouse dose of AAV-mSP-C (Figure 4I). The lack of improvement in MLI using AAV-miR alone illustrates that reducing the accumulation of the dominant-negative form of proSP-C is not sufficient, on its own, to improve lung structure. Conversely, AAV-mSP-C alone improved the MLI, and combined with miRs, it produced a greater effect at the structural level.

**Figure 6:**
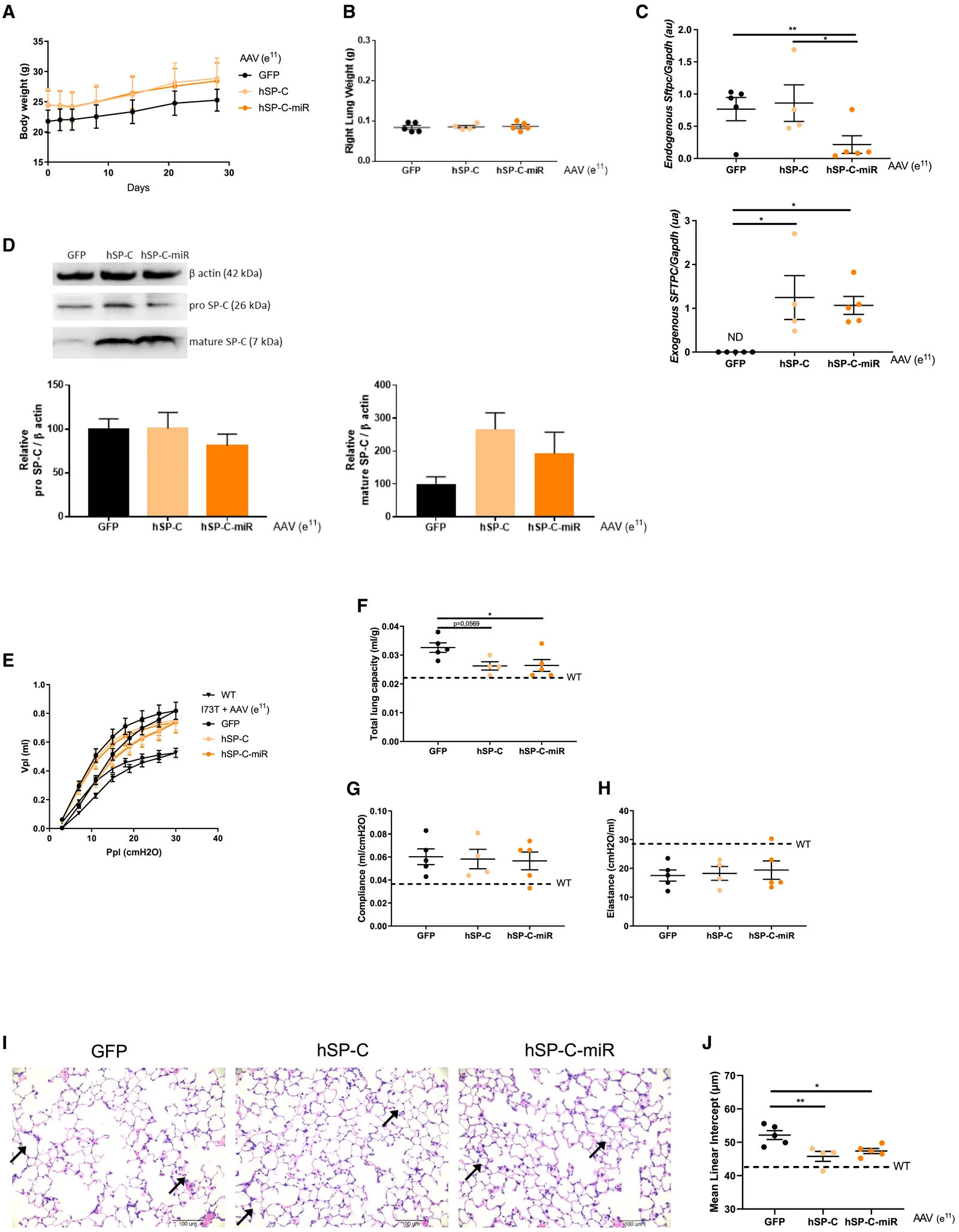
Gene therapy with human SP-C reverses focal airspace enlargement in I73T (A) Evaluation of the body weight of I73T treated with AAV-GFP (*n* = 5), AAV-hSP-C (*n* = 4), and AAV-hSP-C-miR (*n* = 5) at e^11^ vg for 28 days. (B) Graph depicting the right lung weight of I73T treated with AAV-GFP (*n* = 5), AAV-hSP-C (*n* = 4), and AAV-hSP-C-miR (*n* = 5) at e^11^ vg for 28 days. (C) Quantification of *Sftpc* endogenous expression and *SFTPC* exogenous expression in lung tissue homogenate from I73T treated with AAV-GFP (*n* = 5), AAV-hSP-C (*n* = 4), and AAV-hSP-C-miR (*n* = 5) at e^11^ vg for 28 days, relative to *Gapdh*. (D) Representative western blot and quantification of proSP-C and mature SP-C protein expression levels in lung tissue homogenate from I73T treated with AAV-GFP (*n* = 5), AAV-hSP-C (*n* = 4), and AAV-hSP-C-miR (*n* = 5) at e^11^ vg for 28 days, relative to β-actin (not significant). (E) Pressure-volume (PV) curve and body-weight-adjusted PV loop. PV loops are shown as the mean volume of all animals within one group at each pressure point. (F) Total lung capacity (GFP, *n* = 5; hSP-C, *n* = 4; and hSP-C-miR, *n* = 5). (G) Compliance (GFP, *n* = 5; hSP-C, *n* = 4; and hSP-C-miR, *n* = 5, not significant). (H) Elastance (GFP, *n* = 5; hSP-C, *n* = 4; and hSP-C-miR, *n* = 5, not significant). (I) Representative images of lung histology stained with H&E from I73T mice. Focal airspace enlargement (arrows) is diminished in treated lungs. (J) Graph depicting the MLI based on H&E-stained slides from I73T treated with AAV-GFP (*n* = 5), AAV-hSP-C (*n* = 4, ∗∗*p* = 0.0072), and AAV-hSP-C-miR (*n* = 5, ∗*p* = 0.0270) at e^11^ vg for 28 days. Sex of the animals in groups AAV-GFP (*n* = 5, 3 males and 2 females), AAV-hSP-C (*n* = 4, 2 males and 2 females), and AAV-hSP-C-miR (*n* = 5, 2 males and 2 females) are shown. One-way and two-way ANOVA tests were used to determine significance. Data are presented as mean ± SEM.

With these promising results, we designed AAV genomes expressing human SP-C: AAV-hSP-C and AAV-hSP-C-miR. A single oropharyngeal administration of the human version (e^11^ vg/mouse) did not affect survival, body weight, and right lung weight (Figures 4A, 6A and 6B). AAV-hSP-C and AAV-hSP-C-miR expressed exogenous *SFTPC*, and AAV-hSP-C-miR decreased *Sftpc* endogenous expression (Figure 6C). Both AAV-hSP-C and AAV-hSP-C-miR increased mature SP-C protein without inducing an increase in proSP-C protein expression (Figures 6D and S3D). The AAV-hSP-C allowed the production of a mature SP-C without increasing the toxic protein expression of proSP-C caused by the mutant proteins. At the functional level, AAV-hSP-C and AAV-hSP-C-miR improved the total lung capacity (Figure 6F) without affecting other lung function parameters such as the pressure-volume (PV) loop (Figure 6E), compliance (Figure 6G), and elastance (Figure 6H). At the structural level, AAV-mSP-C and AAV-hSP-C-miR reduced focal airspace enlargement (Figure 6I) as quantified by the MLI (Figure 6J).

## Discussion

In this work, we observed focal airspace enlargement associated with the accumulation of proSP-C in I73T mice and showed, for the first time, that a rationally designed AAV6.2FF gene therapy targeting the pathogenic variant of the *Sftpc* gene and toxic protein in a dual approach combining gene replacement with mutant protein knockdown can improve lung function and structure. Here, we show that a single oropharyngeal dose of AAV6.2FF delivering murine *Sftpc* cDNA transgene into I73T mice restored WT *Sftpc* and mature SP-C protein expression and significantly improved lung function and structure. Furthermore, the combinatorial approach (gene replacement and the mutated version silenced by miRNAs) with an AAV-SP-C-miR decreased endogenous *Sftpc* expression, restored WT *Sftpc,* and re-expressed the mature SP-C protein without changing proSP-C protein expression. Using an AAV vector containing the human *SFTPC* alone or combined with miR, similar therapeutic effects were observed. Our findings open new treatment avenues for patients with *Sftpc*/*SFTPC* mutation-associated lung disease.

The KI I73T mutation in C57BL/6n mice results in a decrease in *Sftpc* mRNA and mature SP-C protein expression compared to WT mice. I73T mice demonstrate proSP-C accumulation in AECII cells and focal airspace enlargement without fibrosis. Common markers for proliferation and oxidative stress were studied,^9^^,^^10^ and no difference in expression between I73T and WT mice was observed. The ultrastructure of the apical side of the plasma membrane, lamellar bodies, and surfactant production with the meshwork of tubular myelin produced in the alveolar airspace were altered in I73T compared to WT mice. The observed focal airspace enlargement and proSP-C accumulation are consistent with the previous finding showing that SP-C transits to the cell surface during normal trafficking, but, in the I73T pathogenic variant, it accumulates at the cell surface through both delayed trafficking and active recycling.^11^^,^^12^^,^^13^

Using our I73T mouse model, we evaluated the therapeutic benefits of AAV-based gene therapies to correct the lung phenotype and proSP-C protein accumulation. Our first approach involved gene replacement therapy by administration of an AAV encoding the *Sftpc* cDNA to re-express a healthy version of the murine gene, as previously achieved for SP-B deficiency.^3^ The second approach involved the introduction of a miR155 scaffold encoding three miRNAs targeting the endogenous pathogenic variant of *Sftpc* I73T downstream of the miRNA-resistant *Sftpc* cDNA. The rationale was to adapt the gene therapy to the underlying disease mechanism to remove the toxic dominant-negative proSP-C protein and re-express only a healthy version of the protein. To the best of our knowledge, this is the first example of a dual therapy approach to treat a lung disease caused by a toxic gain-of-function mutation in a single gene. This approach has, however, been shown to be effective at treating a mouse model of alpha-1-antitrypsin (AAT) deficiency when AAV was administered intravenously to target mutant AAT expression in the liver.^14^

AAV6.2FF has the advantage of specifically transducing lung epithelial cells,^15^ particularly AECIIs (approximately 78.88% of AECIIs are transduced), safely and without inducing an inflammatory or immune response on first administration,^3^ As the capsid is the same and only the transgene expressed is different, we did not revisit this experiment in an effort to reduce the animal number used in the study; however, there was no inflammation and no neutrophil infiltration in our H&E- and Sirius red-stained lung sections, nor was there fibrosis development in our study.

We previously documented stable AAV6.2FF-mediated transgene expression in the lungs for up to 200 days and a substantial improvement in survival in a conditional SP-B-knockout (KO) mouse model,^3^ making this vector an ideal candidate for delivering the *Sftpc* transgene either alone or in combination with a miRNA-targeting pathogenic I73T variant of SP-C. One of the advantages of this type of construct is the low risk of insertional mutagenesis due to the persistence of AAV genomes as episomes. However, long-lasting transgene expression only occurs when non-dividing or slowly dividing cell populations are transduced, as previously mentioned. A remaining question is whether one administration is sufficient or whether additional administrations are required to maintain a therapeutic benefit. We are actively investigating this critical aspect of AAV-based gene therapy and have demonstrated success with repeated administrations to sustain the therapeutic efficiency.^16^

Single and combinatorial constructs were designed for murine *Sftpc* and human *SFTPC*; however, the miRNA design in both vectors targets the endogenous pathogenic I73T variant of *Sftpc*. After validation of our constructs *in vitro*, we showed *in vivo* that a single oropharyngeal administration of AAV-SP-C restored *Sftpc* gene expression and mature SP-C protein production, both with the murine and human constructs, and significantly decreased the focal airspace enlargement and ameliorated lung function parameters. Whereas the AAV-miR alone reduced proSP-C protein accumulation in I73T mice, the combined AAV-SP-C-miR (murine/human) led to a decrease in endogenous *Sftpc* expression and restored WT *Sftpc*, leading to re-expression of the mature SP-C protein without significant proSP-C protein modulation. This is consistent with the possibility of distinguishing both endogenous and exogenous gene expression with specific primers, but not the proSP-C protein expression, as the final proteins synthesized are identical. While AAV-SP-C-miR restored the lung structure to a WT state, functional changes were less prominent. However, possible discrepancies have been observed between the morphological and functional lung parameters.^17^ The respiratory mechanics do not always reflect the lung morphological changes in lung diseases, as observed in a study comparing the susceptibilities of different strains of mice to develop emphysema after cigarette smoke exposure^18^ or in a study using a protease model to develop emphysema.^19^ In both of the abovementioned studies, the MLI was significantly increased, but there was no difference in functional parameters. Similar results have shown no correlation between emphysema measured by lung morphology and lung compliance.^20^ Extracellular matrix remodeling has been suggested to interfere with respiratory mechanics so that morphometric parameters are more reliable for detecting the presence of lung injury than functional parameters measured by respiratory mechanics.

While this study demonstrates the therapeutic benefit of AAV-mediated gene therapy in mitigating lung injury in a preclinical model recapitulating focal airspace enlargement and aberrant trafficking and processing of the SP-C proprotein, a limitation is the absence of data on the therapy’s effectiveness against the fibrotic component in *SFTPC* mutation-associated lung disease. This gap underscores the necessity for future evaluations of the described AAV gene therapy in a preclinical disease model prone to developing fibrosis, as demonstrated in Nureki et al.^9^ Such testing in a fibrosis-prone model is crucial to establish the therapy’s efficacy and safety, thereby facilitating successful clinical translation.

Current therapeutic strategies for *SFTPC* mutation-associated lung disease, including anti-inflammatory and anti-fibrotic agents, remain palliative and fail to address the underlying genetic cause.^21^^,^^22^ To address this unmet need, we developed a rationally engineered AAV6.2FF-based gene therapy that combines two mechanistically distinct interventions: (1) miRNA-mediated silencing of the pathogenic variant of *SFTPC* transcripts to eliminate toxic proSP-C aggregates and (2) concurrent delivery of a codon-optimized *SFTPC* transgene to restore functional SP-C expression. In the I73T mouse model, this dual approach corrected the defining functional and histopathological features of mutant SP-C-driven lung injury. While further studies are needed to evaluate efficacy against fibrosis, our findings establish proof of concept for a precision medicine intervention that halts disease progression at its source. Early genetic screening paired with this dual therapy could transform outcomes for patients with inherited *SFTPC*-related lung disorders, shifting treatment paradigms from symptom management to curative repair. Additionally, corticosteroid pulse treatment with methylprednisolone has shown potential to enhance proSP-C processing and reduce toxic protein accumulation by modulating SFTPC mRNA expression and splicing, and combining this with our gene therapy may further improve therapeutic outcomes in *SFTPC* mutation-associated lung disorders.

## Materials and methods

### Institutional approval for animal study

All mouse experiments were approved by the Animal Care and Veterinary Services (ACVS) Committee at the University of Ottawa under the protocol OHRI-1696 and the University of Guelph Animal Care Committee, AUP#4664. All experiments complied with the animal guidelines of the Canadian Council on Animal Care, following the 3Rs compliance and ARRIVE guidelines.^23^

### Animal model

The following procedures were performed at the Ottawa Hospital Research Institute and the University of Ottawa. This study used transgenic C57BL/6n mice containing the I73T-KI mutation in the *Sftpc* gene (I73T mice). These mice were generated by T.E.W.’s lab, and as previously described,^4^ these mice harbor a constitutively expressed pathogenic variant of the *Sftpc* gene. The detailed procedure is provided in supplemental materials and Methods S1.^4^

### RNA extraction and RT-qPCR

The detailed procedure is provided in supplemental materials and Methods S2.

### Immunohistochemistry for proSP-C

The detailed procedure is provided in supplemental materials and Methods S3.

### Protein extraction and western blot

The detailed procedure is provided in supplemental materials and Methods S4.

### MLI measurement

The detailed procedure is provided in supplemental materials and Methods S5.

### AH modified score

Fibrosis was quantified using the AH modified score as previously described^24^ on H&E-stained slides.

### Lung function analysis

The detailed procedure is provided in supplemental materials and Methods S6.

### Lung preparation for transmission electron microscopy

The detailed procedure is provided in supplemental materials and Methods S7.

### Recombinant AAV plasmid generation

The recombinant AAV genomes were designed and produced at the University of Guelph in S.K.W.’s lab. The AAV genome plasmids comprised a composite CASI promoter^25^ consisting of the human cytomegalovirus immediate-early gene enhancer region, the chicken β-actin promoter, and the human ubiquitin C promoter, as well as the woodchuck hepatitis virus post-transcriptional regulatory element (WPRE) and a simian virus 40 polyadenylation sequence downstream of the transgene with flanking AAV2 inverted terminal repeats (ITRs).^15^ The murine *Sftpc* cDNA sequence (GenBank: NM_011359.2_miRNA-resistant) and the human *SFTPC* cDNA sequence (GenBank: NM_001172357.2 transcript variant 3 miRNA-resistant) were synthesized (GenScript) after codon optimization for mouse/human expression using the GenScript codon optimization tool and inserted into the KpnI-XbaI site of the AAV genome. The engineered pre-miRNA sequence targeting endogenous murine SP-C small interfering RNA (siRNA) was designed by inserting modified versions of three siRNAs targeting murine SP-C into the miR155 backbone. The following antisense and sense sequences targeting nucleotides 14–35, 299–320, and 413–434 of the *Sftpc* cDNA were inserted into the miR155 scaffold of the BLOCK-iT Pol II miR RNAi Expression Vector Kit (Invitrogen), antisense 14–35 bp pre-miRNA ctctccatcaggacctctttgc and sense strand GTAAAGAGGctgatggagag, antisense 299–320 bp pre-miRNA tggtagtcatacacaacgatgc and sense strand GTATCGTTGtatgactacca, and antisense 413–434 bp pre-miRNA tggaagttctggagttttctag and sense strand CTAGAAAACcagaacttcca to create three tandem sequences comprising a 5′ miR flanking region-5′G + antisense target sequence-loop sequence-sense Δ2nt target sequence-3′ miR flanking region. This triple pre-miRNA was then cloned immediately downstream of the miRNA-resistant *Sftpc* or *GFP* gene. The miRNAs were identified and screened as previously described.^26^

### AAV6.2FF vector production

The AAV6.2FF vectors were produced in adherent human embryonic kidney (HEK)293 cells and purified as described previously.^27^^,^^28^ Vector genomes were quantified using TaqMan qPCR assay by targeting the ITR sequence of the AAV2 genome using the following primers: forward, 5′-GGA ACC CCT AGT GAT GGA GTT-3′; reverse, 5′-CGG CCT CAG TGA GCG A-3′; and probe, 5′-FAM-CAC TCC CTC TCT GCG CGC TCG-BHQ-3′.

### *In vitro* cell culture and transfection

The detailed procedure is provided in supplemental materials and Methods S8.

### Oropharyngeal administration

The detailed procedure is provided in supplemental materials and Methods S9. Mice received the AAV-GFP (control), AAV-mSP-C, AAV-hSP-C, AAV-mSP-C-miRNA, and AAV-hSP-C-miRNA in a final volume of 50 μL. Mice were injected with e^11^–e^12^ vg/mouse depending on the experimental design.

### Immunofluorescence

The detailed procedure is provided in supplemental materials and Methods S10.

### Statistical analysis

All statistical analyses were performed with GraphPad Prism 8.0. Grubbs’ test determined the presence of statistical outliers. Data were presented as means ± SEM. Differences between 2-member groups were evaluated by the two-tailed Student’s *t* test. Differences between ≥3-member groups were evaluated by ordinary one-way ANOVA with Tukey’s or Dunnet’s multiple comparisons post hoc test or two-way ANOVA. Kaplan-Meier survival curves were analyzed with the log rank, Mantel-Cox test. *p* ˂ 0.05 was considered significant and depicted as follows: ∗*p* ˂ 0.05, ∗∗*p* ˂ 0.01, ∗∗∗*p* ˂ 0.001, and ∗∗∗∗*p* ˂ 0.0001.

## Data and code availability

All datasets generated for this study are included in the article/supplemental information.

## Acknowledgments

The authors thank all the ACVS veterinary and technical staff at the University of Ottawa; all the staff at the University of Ottawa Histology Core, especially Zaida Ticas and Sharlene Faulkes; and Yves de Repentigny from the Dr. Rashmi Kothary lab at the Center for Neuromuscular Disease, Cellular and Molecular Medicine Department, Regenerative Medicine Program, for his help with TEM lung image acquisition. This work was supported by funding from the European Respiratory Society and the European Union’s H2020 research and innovation program under the Marie Sklodowska-Curie grant agreement 847462 to P.B. Additionally, this work was supported by CIHR (PJT-166009) and Collaborative Health Research Project (NSERC partnered) (433339) grants to S.K.W. and B.T.

## Author contributions

P.B. designed, generated, and analyzed all experiments, performed *in vitro* studies, contributed to the study design, and drafted the manuscript. E.M. and T.E.W. provided the I73T mouse model. Y.P. and S.K.W. designed, produced, and purified the AAV vectors and conducted the *in vivo* experiments relating to IF. P.B., K.N., and L.X. maintained and genotyped the transgenic mice, while P.B., K.N., L.X., and A.V. performed the *in vivo* experiments. P.B. and K.N. monitored respiratory distress and tracked body weight in the survival studies. FlexiVent data collection and lung harvesting for histology were performed by P.B., K.N., S.Z., A.V., and L.X. P.B., K.N., and C.C.-D. conducted qPCR, MLI, western blotting, and fibrosis scoring. A.A.V. performed fluorescent immunohistochemistry and reviewed and edited the manuscript. S.K.W., C.C.-D., S.S., and B.T. provided critical feedback and suggestions during manuscript preparation.

## Declaration of interests

S.K.W. and B.T. are the co-founders and chief scientific officer and chief medical officer of Inspire Biotherapeutics, a preclinical, pre-revenue-stage gene therapy company developing AAV-based therapies for monogenic lung diseases. S.K.W. and B.T. are inventors on issued patents in Canada and the US for the AAV6.2FF capsid, which are owned by the University of Guelph and licensed to Inspire Biotherapeutics. The authors are solely responsible for the content; it does not represent the opinion of the European Respiratory Society (ERS) or the European Commission. ERS and the EU Commission are not responsible for any use that might be made of data appearing therein.

## Supplemental information

Document S1. Figures S1–S4, Table S1, and Methods S1–S10

Document S2. Article plus supplemental information
