# Supplementary material for: Lung-tropic dual AAV-SP-C and microRNA gene therapy attenuates lung injury in mutant Sftpc mice
Source: Mol Ther. 2025 Dec 24;34(4):1963–75. doi: 10.1016/j.ymthe.2025.12.049 (PMC13069285; doi:10.1016/j.ymthe.2025.12.049)
Supplement: Supplementary file 1 [file mmc1.pdf]

YMTHE, Volume *34*

Supplemental Information

Lung-tropic dual AAV-SP-C and microRNA gene therapy attenuates lung injury in mutant *Sftpc* mice

Pauline Bardin, Liqun Xu, Yanlong Pei, Adithya Achuthan Vineeth, Kennedy Nangle, Shumei Zhong, Chanèle Cyr-Depauw, Arul Vadivel, Emily Martin, Timothy E. Weaver, Sneha Sitaraman, Sarah K. Wootton, and Bernard Thébaud

Supplemental Methods

SM1. Animal model

Mice were housed at the University of Ottawa ACVS facility on a 12h/12h off cycle and at room temperature (RT) of 21°C with a regular mouse chow, in an open cage system. Up to 4 animals were housed per cage and grouped with littermates and by sex after weaning, with numerical ID. To confirm mouse genotype, we used previously established protocols.[1] Genotyping PCR was performed using DNA extracted from samples obtained from clipped ears or tails using the AccuStart II Mouse Genotyping Kit (Quanta Biosciences) according to the manufacturer's instructions. Primers used for genotyping: primers P1 (exon 1): TGG ACA TGA GTA GCA AAG AGG TC, P2-625 (BGH pA): CCA TCT GTT GTT TGC CCC TC and P4 (intron 1): ATC CTA AAA GCC CAA TCC TAA GC.

SM2. RNA extraction and RT-qPCR

30 mg of right lung snap frozen was weighed, cut, and placed in a 2 mL tube kept on ice. Mechanical and chemical digestion using a 5 mm stainless steel bead and lysis buffer (RNeasy Plus mini kit #74136, Qiagen) was done using the Tissue-Lyser II machine (Qiagen) for 2x 1 min 30 sec at 25 Hz. Beads were removed, and the homogenized tissue was used to extract the RNA following the extraction protocol according to the manufacturer's instructions. The same protocol was used to extract RNA from cells. RNA was eluted with 40 µL of sterile RNase-free water. The concentration and quality of the RNA were evaluated using a Nanodrop One/OneC Microvolume UV-Vis Spectrophotometer (Fisher) based on absorbance at 260 nm and RNA extracts stored at -20°C. The sequences were reverse transcribed with the iScript™ Reverse Transcription Supermix Kit (#1708841, Biorad) using 500 ng of RNA. qPCR was performed using CFX384™ Optics Module (Biorad) and TaqManTM technology. Primer and probe sequences used for RT-qPCR analysis are detailed in Table S1. For relative quantification, RNA levels were calculated using the $2^{-\Delta Ct}$ method and normalized to the expression levels of *Gapdh/GAPDH*. Each sample was assessed in triplicate.

SM3. Immunohistochemistry for proSP-C

Paraffin-embedded tissue blocks were sectioned at 4 µm, and IHC staining was performed using the Leica Bond™ system using a modification of protocol F that eliminates the post-primary step when using rabbit antibodies on mouse tissue. Sections stained were pre-treated using heat-mediated antigen retrieval with citrate buffer (pH 6.0, epitope retrieval solution 1) or EDTA buffer (pH 9.0, epitope retrieval solution 2) for 20 mins. The sections were then incubated using the indicated dilutions for 30 mins at RT and detected using an HRP conjugated compact polymer system. Slides were then stained using DAB as the chromogen, counterstained with Hematoxylin, mounted, and coverslipped.

SM4. Protein extraction and Western blot

Snap-frozen right lung samples were cut and placed in a 2 mL tube to be mechanically and chemically digested using a 5 mm stainless steel bead in a 700 µL of phosphate-buffered saline (PBS), 1% Protease

inhibitor (PI78430, Fisher), and 1X Phosphatase Inhibitor (PI78420, Fisher). Tubes were placed on ice and homogenized using the TissueLyser II machine (Qiagen) for 2x 1 min at 25 Hz. The supernatant was removed and placed in a new vial for storage at -20°C. Total protein concentration was evaluated with a PierceTM BCA Protein Assay Kit (Thermo Fisher Scientific). Protein dosage was done using a Bio-Rad Quick Start Bradford 1x Dye Reagent Each sample followed the protocol extraction according to the manufacturer's instructions (# 500-0205 BioRad). Protein concentrations were quantified using the Gen5 software at 595 nm. Next, 30 μg of total protein extract was reduced and size-separated on Tris-Tricine gels (Invitrogen), transferred to nitrocellulose membranes (Biorad), blocked in TBS-T milk, and incubated with specific primary antibodies against mature SP-C (#WRAB-76694, Seven Hills Bioreagent, 1:1,000), proSP-C (#WRAB-9337, Seven Hills Bioreagent, 1:1,000) and βactin (#8H10D10, Cell signaling/new england biolabs, 1:1,000). The proteins of interest were detected, imaged, and quantified (Chemidoc; Bio-Rad).

SM5. Mean Linear Intercept measurement

Each mouse lungs were perfused with 10% neutral buffered formalin (NBF) at a constant pressure of 20cm H_2O for a duration of 3 minutes and then transferred into a tube containing 10% NBF where fixed for 48 hours and then embedded in paraffin. Paraffin-embedded tissue blocks were sectioned at 4 μm and stained with hematoxylin and eosin (H&E), by the Louise Pelletier Histology Core at the University of Ottawa. Slides were scanned using the Axio Scan.Z1 (Zeiss, Oberkochen, Germany). The mean linear intercept (MLI) was estimated with Metamorph software (Quorum Technologies, Puslinch, ON) using a 155.34 μm line as described previously.[2,3] A total of 200 randomly selected 500 μm × 500 μm fields of view were assessed in each lung.

SM6. Lung function analysis

Lung function was measured following the generation of individual pressure-volume (PV) curves for each mouse as previously described.[4,5] Briefly, mice were euthanized with an intraperitoneal injection of Euthanyl (Pentobarbital Sodium Injection; Bimeda-MTC Animal Health Inc). Within 10-15 mins, PV curves were obtained using a small animal mechanical ventilator (FlexiVent, Scireq). A cannula attached to the FlexiVent was secured to the trachea of euthanized animals in a supine position. The lungs were inflated with regular increasing pressure intervals to a maximum of 30 cmH_2O. Lungs were subsequently deflated with regular decreasing pressure intervals to obtain PV curves. All data were obtained using the flexiWare version 7.0 (Scireq) software. PV curves were normalized to the body weight of each animal. Total lung capacity (TLC), residual volume (RV), %v10, compliance, and elastance were extracted from the pressure-volume curves.[6] Briefly, the TLC is the volume at the defined maximal pressure of 30 cmH2O. %v10 quantifies the shape of the deflation limb and decreases with surfactant inhibition and is calculated as $V_{10} \div V_{30}$. The RV is the residual air trapped in the alveoli at maximal expiration at the end of the deflation curve. The compliance is the slope at any linear region of the

deflation limb and was calculated by the ratio in the change in volume between 10 cmH2O and 7 cmH2O (ΔV) over the change in pressure (ΔP) and its reciprocal parameter, elastance.

SM7. Lung preparation for Transmission Electron Microscopy

C57BL6/n 8-10-week-old mice (WT n=2; I73T n=2) were anesthetized and perfused transcardially with 20 ml of PBS followed by 40 ml of Karnovsky's fixative. The left lungs were extracted and kept fixed overnight at 4ºC. For our analysis, 3 segments of 1 mm of length were collected from each lung. Specimens were washed in 0.1 M sodium cacodylate and post-fixed with 1% osmium tetroxide in 0.1 M sodium cacodylate buffer. Samples were completely dehydrated and were embedded in liquid Spurr resin for 3 days at RT before being polymerized overnight at 70ºC. Ultrathin sections (80 nm) were collected onto 200-mesh copper grids and stained with 2% aqueous uranyl acetate and with Reynold's lead citrate. Electron micrographs of the AECIIs were taken with a JEOL Transmission Electron Microscope (JEM-1400Plus, Gatan OneView digital camera,) operated at a voltage of 80 kV at the University of Ottawa. More than 100 electron micrographs were examined at different magnifications for WT and I73T.

SM8. *In vitro* cell culture and transfection

HEK293 cells (HEK293; ATCC® CRL-1573) were maintained in high glucose DMEM (HyClone; SH30022.01) with 10% cosmic calf serum (HyClone; SH30087), 2 mM L-glutamine (HyClone; SH3003401), and 1% pen-strep (HyClone; SV30010). Murine lung epithelial 12 cells (MLE12; ATCC® CRL-2110) were maintained in DMEM/F12 1:1 media including HEPES (HyClone; SH30261) with the addition of 2% fetal bovine serum (FBS; HyClone; SH30088), insulin (0.005 mg per mL)-transferrin (0.01 mg per mL)-selenium 30 nM (Gibco; 41400045), 10 nM hydrocortisone (Sigma; H6909), 10 nM β-estradiol (Sigma; E2758), 2 mM L-glutamine (HyClone; SH3003401), and 1% pen-strep (HyClone; SV30010). At 80% confluency, HEK293 and MLE12 were transduced using different AAV constructions at different multiplicity of infection (MOI) 5,000-20,000[7] for 96h.

SM9. Oropharyngeal administration

Treatment was randomly assigned to 8-10-week-old mice. Animals were monitored consistently throughout the study and weighed following treatment administration. Mice were anesthetized using isoflurane (Fresenius Kabi). The mouse respiration was monitored visually, mice were tachypneic and tachycardic. When deeply anesthetized (breathing slowly from the abdomen) the mice were placed on vertical support (Harvard Apparatus) and suspended by their upper incisors using surgical thread. The ventral side of the mouse was positioned facing away from the operator and the tongue was gently pulled out using forceps, allowing to keep the trachea open. The 50 µL of the AAV contained in a pipette, was placed in the oral cavity of the mice, and the volume was administered. After 3-4 breaths, the mice were removed from the apparatus and left to recover in an incubator for 15-30 mins. Mice were monitored for a few hours following the procedure to ensure proper recovery.

SM10. Immunofluorescence

The lung sections were stained with primary rabbit anti-proSP-C antibody EMD; AB3786, 1:250) for 48h at 4°C. The sections were then washed and incubated with FITC-conjugated goat anti-rabbit antibodies (1:500). After additional washing steps, the sections were mounted using a DAPI-containing mounting buffer. DAPI (blue) was used to label nuclei, with an exposure time of 1200 ms for all DAPI images and 3500 ms for all FITC images.

Table S1. Primer and probe sequences and assay IDs used for RT-qPCR analysis

Name		Reference
Sftpc	endogenous	Mm.PT.58.9922071, exon 1-2
	exogenous	Forward: CCGAAATGGTGTTGGAAATGT; probe: CAATAGGAGCTC CTGAGACCCAAA; reverse: ATAGTATCAGCACGCTCAGAAG
SFTPC	exogenous	Forward: GTCTCCACATGAGCCAGAAA; Probe: CACACGGAGATG GTTCTGGAAATGTCT; Reverse: ATACTGAAAGTGGCAGTGGTAA
Tgfb1		Mm01178820_m1
Col1a1		Mm00801666_g1
Col1a2		Mm00483888_m1
Atf6		Mm01295319_m1
Gapdh		Mm99999915_g1
GAPDH		Hs02786624_g1

Supplemental Figures

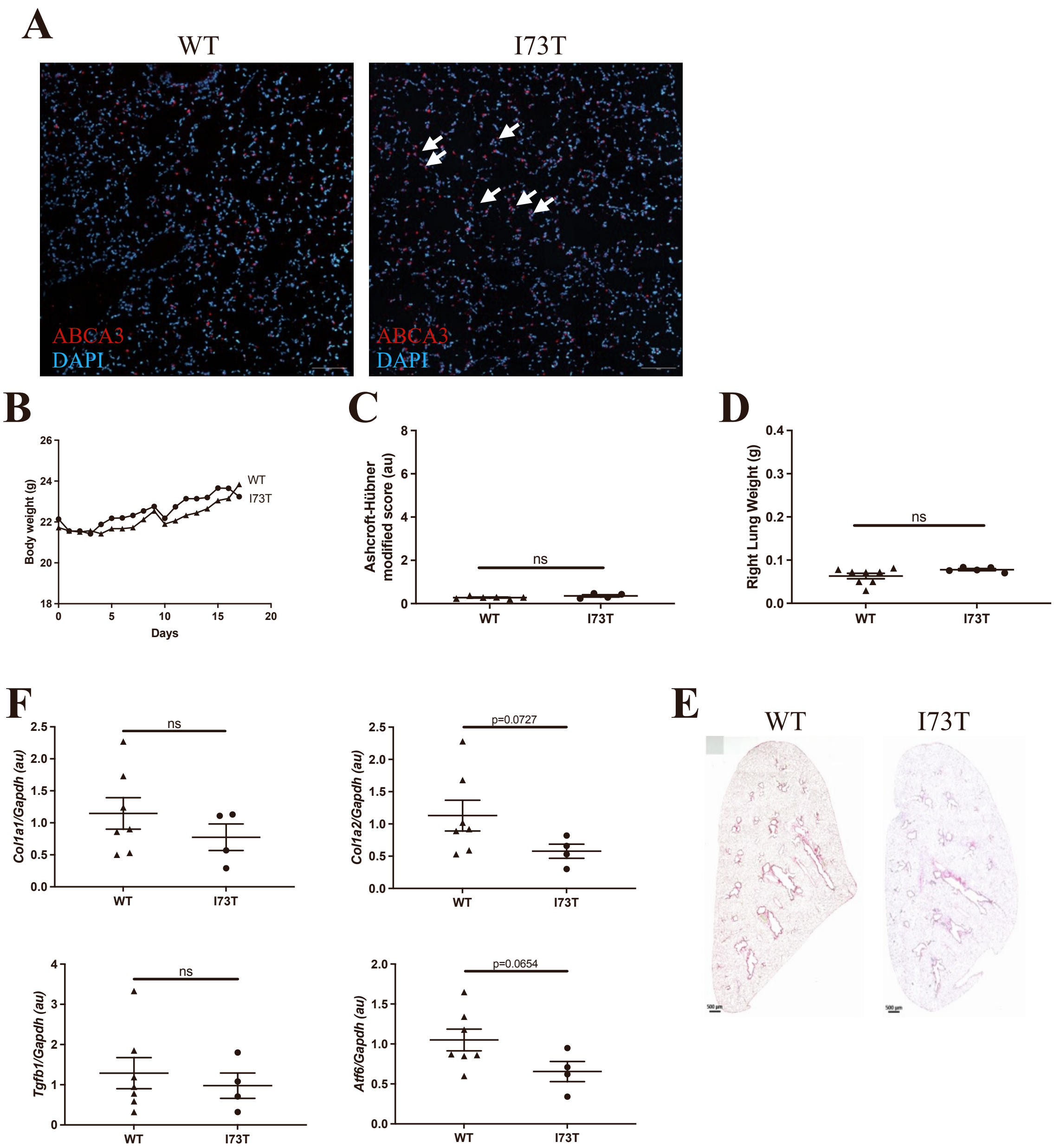

Figure S1. I73T knock-in mouse model characterization. (A) Representative fluorescent immunohistochemistry images showing coexpression of ABCA3 (red) and DAPI (blue) in the airway epithelium of WT (left) and I73T (right) mouse lungs. Scale bars: 100 µm. (B) Evaluation of the body weight of WT (n=8) and I73T (n=4) mice over 17 days. (C) Graph depicting the AH modified score (WT, n=6, I73T, n=4, not significant). (D) Graph depicting the right lung weight (WT, n=8; I73T, n=5, not significant). (E) Sirius red stained lung sections from WT and I73T mice were visualized under bright field. Scale bars, 500 µm. (F) Quantification of *Tgfb1* (p=0.6790), *Atf6* (p=0.0654), *Col1a1* (p=0.3159), *Col1a2* (p=0.0727) expressions in lung explant from WT (n=7) and I73T (n=4) mice, relative to *Gapdh*. An unpaired t-test was used to determine the significance between the two conditions.

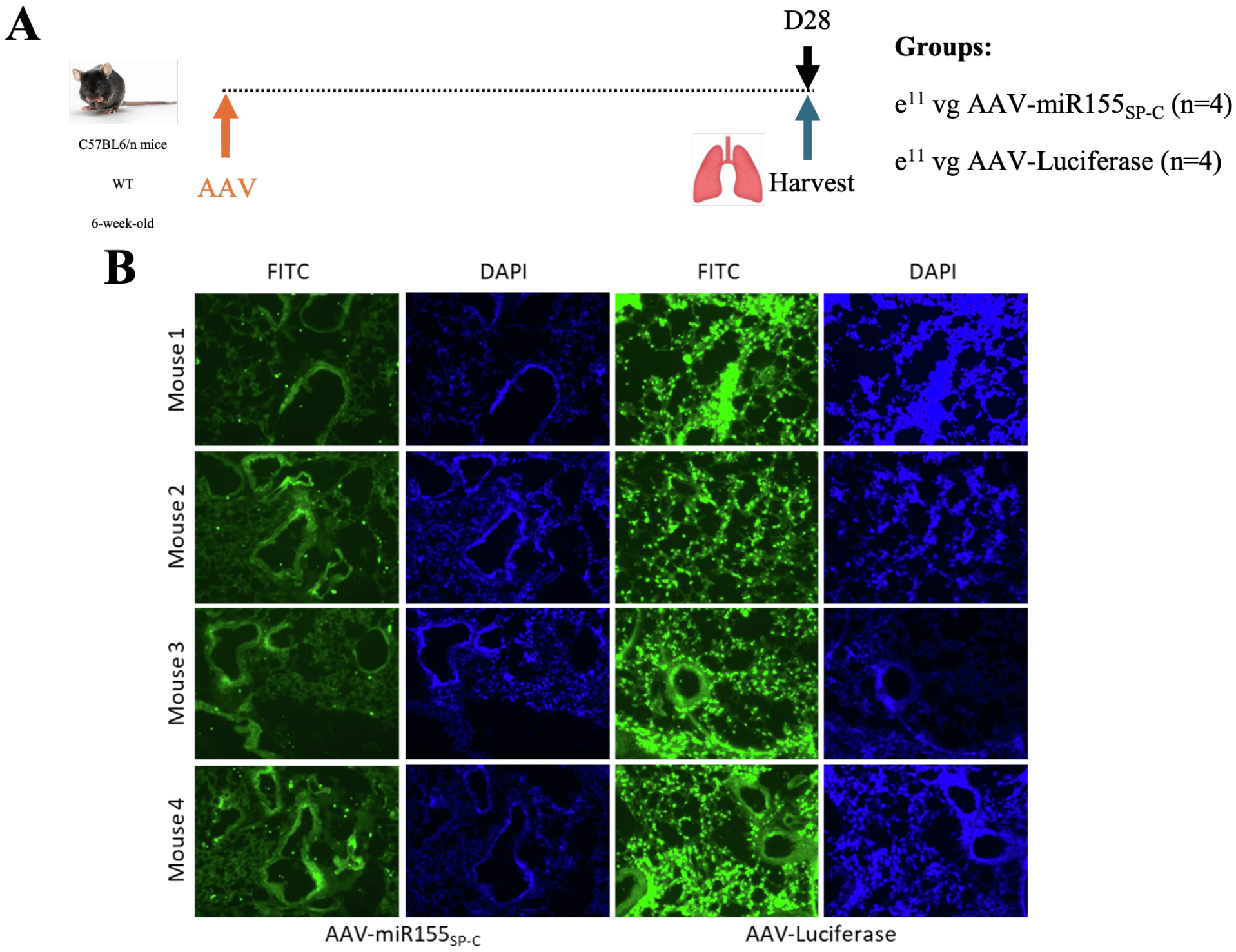

Figure S2. AAV-expressed miR155 against murine SP-C significantly reduces endogenous proSP-C protein expression.

(A) Experimental design. (B) Immunofluorescence staining was performed on lung tissue cryosections from the same mice that were treated with either AAV-GFP-miR155$_{SP\text{-}C}$ or AAV-luciferase for 28 days.

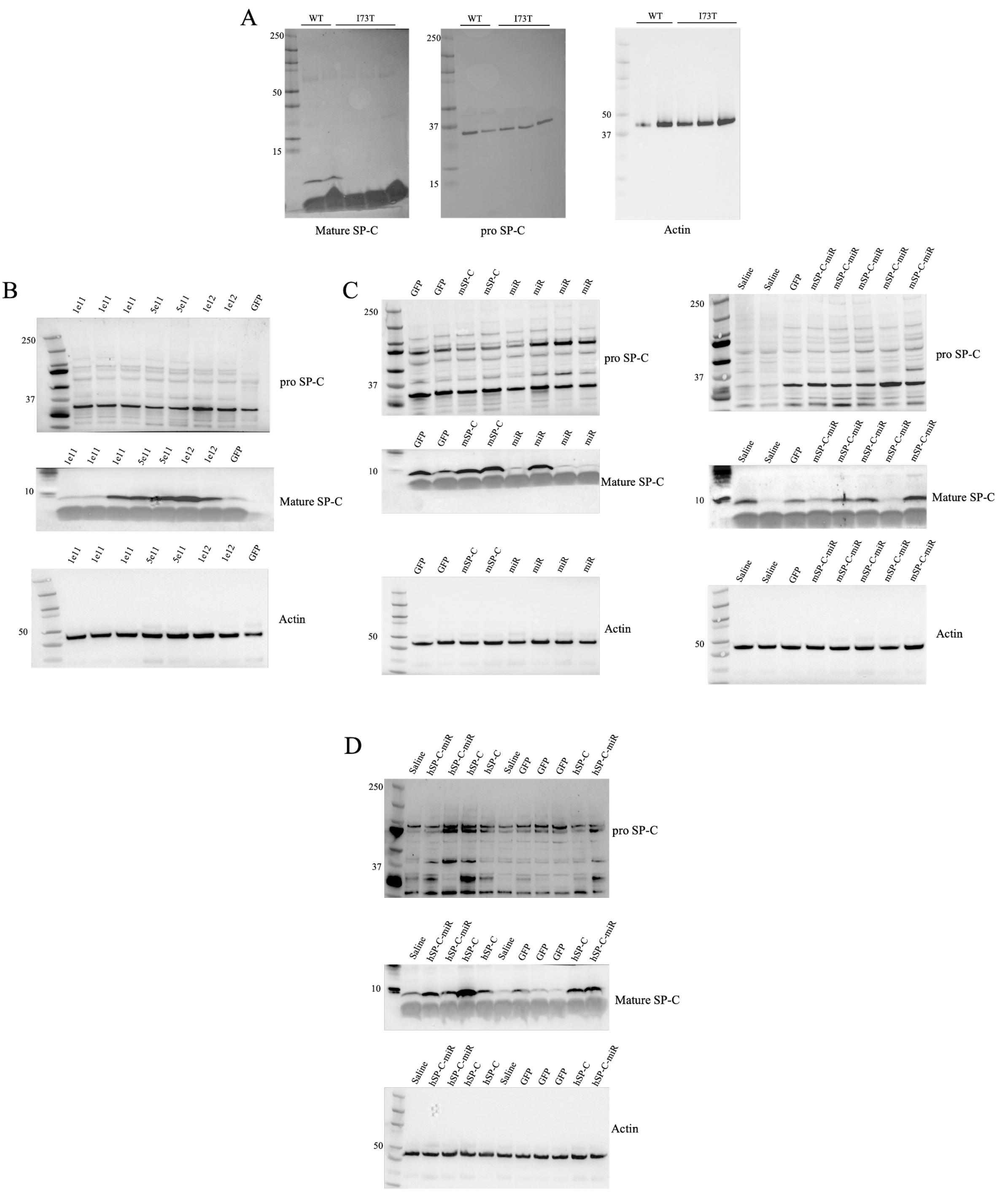

Figure S3. Full unedited blots from figures 1,4,5, and 6. (A) Full unedited blot from figure 1D. (B) Full unedited blot from figure 4D. (C) Full unedited blot from figure 5C. (D) Full unedited blot from figure 6D.

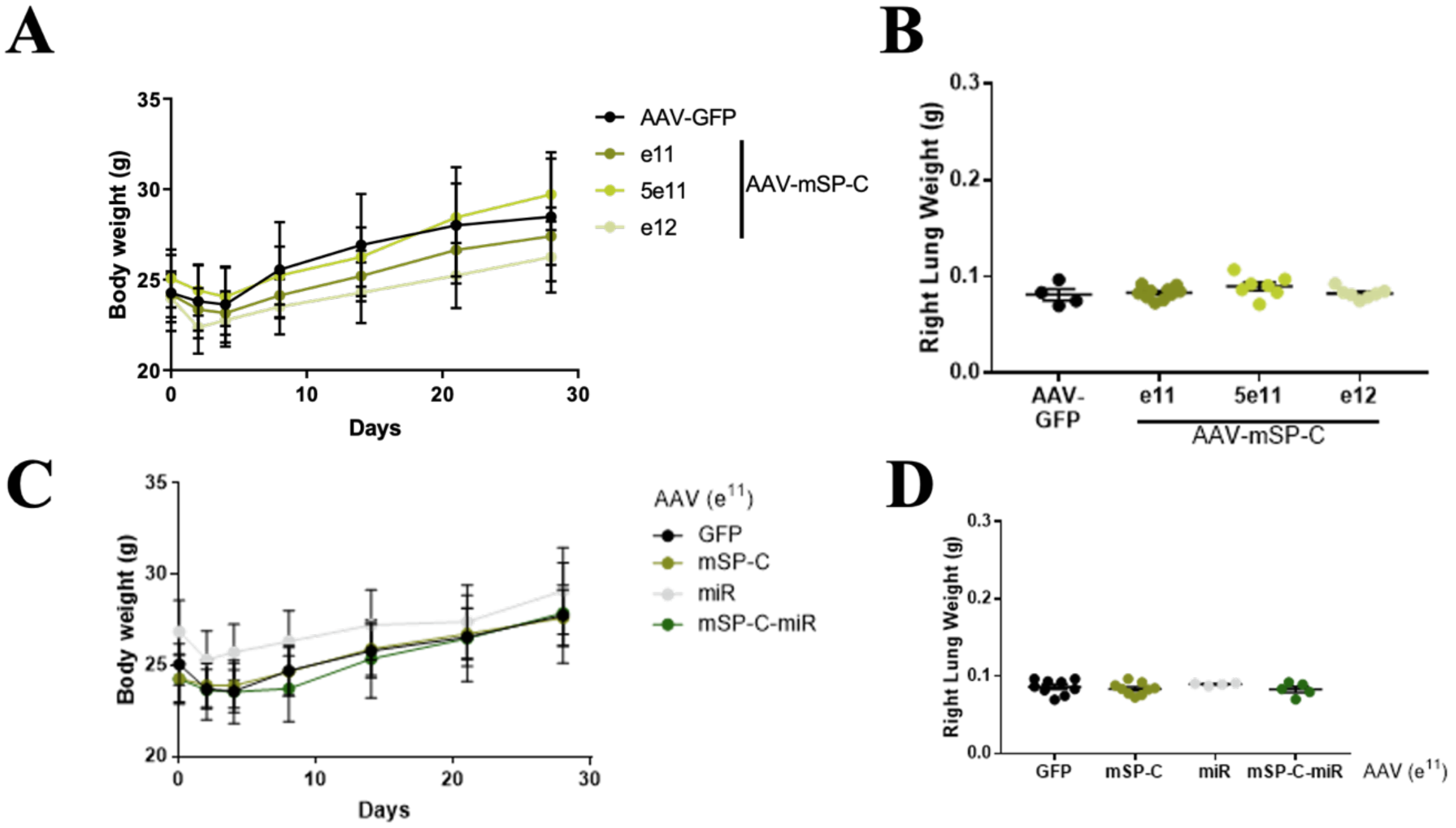

Figure S4. Body and right lung weights (A) Evaluation of the body weight of I73T treated with AAV-GFP (n=4), AAV-mSP-C at different concentrations e^{11}vg (n=8), 5e^{11}vg (n=7) and e^{12}vg (n=7) for 28 days. (B) Graph depicting the right lung weight of I73T treated with AAV-GFP (n=4), AAV-mSP-C at different concentrations e^{11}vg (n=8), 5e^{11}vg (n=7) and e^{12}vg (n=7) for 28 days. (C) Evaluation of the body weight of I73T treated with AAV-GFP (n=9), AAV-mSP-C (n=9), AAV-miR (n=4), AAV-mSP-C-miR (n=5) at e^{11}vg for 28 days (right). (D) Graph depicting the right lung weight of I73T treated with AAV-GFP (n=9), AAV-mSP-C (n=9), AAV-miR (n=4), AAV-mSP-C-miR (n=5) at e^{11}vg for 28 days (right).

References

1. Tang, X., Wei, W., Sun, Y., Weaver, T.E., Nakayasu, E.S., Clair, G., Snowball, J.M., Na, C.-L., Apsley, K.S., Martin, E.P., Kotton, D.N., et al. (2024). EMC3 regulates trafficking and pulmonary toxicity of the SFTPCI73T mutation associated with interstitial lung disease. The Journal of Clinical Investigation. 10.1172/JCI173861.
2. Thebaud, B., Ladha, F., Michelakis, E.D., Sawicka, M., Thurston, G., Eaton, F., Hashimoto, K., Harry, G., Haromy, A., Korbutt, G., and Archer, S.L. (2005). Vascular endothelial growth factor gene therapy increases survival, promotes lung angiogenesis, and prevents alveolar damage in hyperoxia-induced lung injury: evidence that angiogenesis participates in alveolarization. Circulation *112*, 2477-2486. 10.1161/CIRCULATIONAHA.105.541524.
3. Cyr-Depauw, C., Hurskainen, M., Vadivel, A., Mizikova, I., Lesage, F., and Thebaud, B. (2021). Characterization of the innate immune response in a novel murine model mimicking bronchopulmonary dysplasia. Pediatr Res *89*, 803-813. 10.1038/s41390-020-0967-6.
4. Avery, M.E., and Mead, J. (1959). Surface properties in relation to atelectasis and hyaline membrane disease. AMA J Dis Child *97*, 517-523. 10.1001/archpedi.1959.02070010519001.
5. Mitzner, W., Johnson, J.W., Scott, R., London, W.T., and Palmer, A.E. (1979). Effect of betamethasone on pressure-volume relationship of fetal rhesus monkey lung. J Appl Physiol Respir Environ Exerc Physiol *47*, 377-382. 10.1152/jappl.1979.47.2.377.
6. Limjunyawong, N., Fallica, J., Horton, M.R., and Mitzner, W. (2015). Measurement of the pressure-volume curve in mouse lungs. J Vis Exp, 52376. 10.3791/52376.
7. Pillay, S., Meyer, N.L., Puschnik, A.S., Davulcu, O., Diep, J., Ishikawa, Y., Jae, L.T., Wosen, J.E., Nagamine, C.M., Chapman, M.S., and Carette, J.E. (2016). An essential receptor for adeno-associated virus infection. Nature *530*, 108-112. 10.1038/nature16465.
